# 12‑weeks fisetin supplementation and interval resistance with aerobic training: changes in Maresin‑1 and inflammatory markers in men with obesity: a randomized controlled trial

**DOI:** 10.1080/15502783.2026.2679718

**Published:** 2026-05-31

**Authors:** Mehran Alipour, Ayoub Saeidi, Keyvan Hejazi, Ismail Laher, Hassane Zouhal

**Affiliations:** a Department of Physical Education and Sport Sciences, Faculty of Humanities and Social Sciences, University of Kurdistan, Sanandaj, Kurdistan, Iran; b Department of Exercise Physiology, Faculty of Sport Sciences, Hakim Sabzevari University, Sabzevar, Iran; c Department of Anesthesiology, Pharmacology and Therapeutics, The University of British Columbia, Vancouver, Canada; d Faculty of Medical Sciences, UM6P Hospitals, Mohammed VI Polytechnic University, Benguerir, Morocco; e Institut International des Sciences du Sport (2I2S), France

**Keywords:** Combined training, fisetin, inflammation, Maresin-1, obesity

## Abstract

**Background:**

Obesity is characterized by low‑grade chronic inflammation and impaired insulin sensitivity. Maresin‑1 (MaR1), a specialized pro‑resolving mediator, plays a critical role in terminating inflammation and supporting metabolic homeostasis; however, interventional data in humans remain scarce. This study examined whether fisetin supplementation augments the effects of concurrent interval resistance–aerobic training on Maresin‑1, pro‑inflammatory markers, and insulin resistance in obese men.

**Methods:**

In a 12‑week parallel‑group randomized controlled trial, 44 obese adult males (BMI > 30 kg/m²) completed one of four interventions: control-placebo (CP), fisetin (F) (200 mg/day), training-placebo (TP), or training-fisetin (TF). Training comprised eight resistance exercises at 60% 1RM with active rest followed by progressive aerobic bouts (50%–70% HRmax). Anthropometric and biochemical parameters, including plasma Maresin‑1, interleukin-6 (IL‑6), tumor necrosis factor-alpha (TNF‑α), fasting blood glucose (FBS), insulin, and HOMA‑IR, were assessed pre‑ and post‑intervention.

**Results:**

Significant group × time interactions were observed for Maresin‑1 (*p* = 0.034), IL‑6 (*p* = 0.001), TNF‑α (*p* = 0.001), FBS (*p* = 0.001), insulin (*p* = 0.001), and HOMA‑IR (*p* = 0.001). Maresin‑1 increased in the TP (*p* = 0.001) and TF (*p* = 0.001) groups. IL‑6 decreased in T (*p* = 0.006), TF (*p* = 0.001), and F (*p* = 0.013) groups. TNF‑α decreased in all intervention groups (F, TP, and TF) (*p* = 0.002). FBS, insulin, and HOMA‑IR decreased significantly in all active arms (*p* = 0.003), with the greatest reductions in the TF group.

**Conclusion:**

Twelve weeks of concurrent interval resistance–aerobic training, especially when combined with fisetin, improved inflammatory resolution (↑Maresin‑1, ↓IL‑6, and ↓TNF‑α) and metabolic control (↓FBS, ↓insulin, and ↓HOMA‑IR) in obese men. The synergy between exercise‑induced adaptations and fisetin’s anti‑inflammatory properties offers a promising non‑pharmacological strategy for mitigating obesity‑related metabolic risk.

## 1. Introduction

Obesity is a global epidemic and a major risk factor for metabolic syndrome, type 2 diabetes, and cardiovascular disease [1]. A core pathophysiological feature of obesity is low‑grade chronic inflammation driven by adipose tissue dysfunction; adipocytes and infiltrating macrophages secrete proinflammatory cytokines such as tumor necrosis factor-alpha (TNF‑α) and interleukin-6 (IL‑6), which contribute to insulin resistance, lipid metabolic disturbances, and fibrotic processes [2]. Although molecular understanding has advanced considerably, direct human evidence on changes in pro‑resolving mediators and the capacity to modulate them remains limited. Among these mediators, Maresin‑1 (MaR1), a specialized pro‑resolving mediator (SPM), plays a central role in terminating inflammation, promoting tissue repair, and regulating local immune responses [3,4]. Preclinical data indicate that reduced MaR1 production or impaired MaR1 pathways can exacerbate adipose tissue inflammation and compromise insulin sensitivity; yet randomized interventional studies targeting MaR1 in obese human populations are scarce [5,6].

Beyond pharmacological approaches, non‑pharmacological strategies, particularly dietary modification, use of natural supplements, and structured physical activity, are important for attenuating systemic inflammation and improving insulin sensitivity [7,8]. Combined exercise training programs that integrate resistance training with aerobic training simultaneously increase muscle mass, enhance mitochondrial oxidative capacity, and reduce visceral adiposity, thereby activating anti‑inflammatory pathways and improving metabolic function [9,10]. Nonetheless, individual responses to exercise vary, and the magnitude of inflammatory improvement may depend in part on an individual’s capacity to generate SPMs (Specialized Pro-resolving Mediators) and on their adipokine profile; consequently, pairing exercise with a targeted nutritional intervention could produce synergistic benefits.

Fisetin, a naturally occurring flavonoid, possesses antioxidant, anti‑inflammatory, and senolytic properties [11,12]. In cellular and animal models, fisetin has been shown to inhibit inflammatory signaling pathways (including NF‑κB), reduce oxidative stress, and shift macrophage polarization toward reparative phenotypes; there is also preliminary evidence that fisetin may support the production or preservation of SPMs [13,14]. However, human data, particularly randomized controlled trials examining the effects of fisetin on MaR1, proinflammatory cytokines, and insulin resistance in obese populations are very limited. Combining fisetin supplementation with a structured exercise regimen may therefore target multiple complementary mechanisms: lowering systemic oxidative and inflammatory burden, improving mitochondrial and muscle function, and modifying adipocyte–immune interactions in ways that could increase MaR1 and decrease IL‑6 and TNF‑α.

Accordingly, the central scientific question is whether fisetin supplementation can potentiate the beneficial effects of concurrent interval resistance–aerobic training on the inflammatory profile and pro‑resolving mediators such as MaR1 and whether such changes are associated with improvements in insulin resistance in obese men. The present study was designed to address this question by evaluating the effects of a 12‑week program of concurrent interval resistance–aerobic training combined with fisetin supplementation on MaR1, IL‑6, TNF‑α, and insulin resistance in men with obesity.

## 2. Methods

### 2.1. Participants and ethics approval

This randomized controlled trial adopted a parallel-group design and initially recruited 107 adult males with obesity who volunteered to participate. Following eligibility screening, 60 individuals meeting all inclusion criteria were enrolled in the study.

The inclusion criteria specified that participants must present a body mass index (BMI) exceeding ≥ 30 kg/m², and participants were included based on a body fat percentage (PBF) > 25%, ensuring that the high BMI was reflective of adiposity rather than lean muscle mass. All participants were classified as sedentary or inactive for at least six months prior to the study.

Participants were required to report no engagement in structured physical activity and abstain from alcohol consumption during the 6 months preceding enrollment. The exclusion criteria encompassed any physical limitations or joint disorders, endocrine, metabolic, or cardiovascular disease, as well as the use of prescription medications or nutritional supplements known to influence muscle or adipose tissue metabolism. Additionally, individuals consuming over-the-counter products containing caffeine, protein, or similar bioactive compounds were excluded.

A familiarization session was conducted one week prior to initiating the intervention protocols. During this session, the study design and procedures were explained, and the participants were also familiarized with the correct technique for each resistance exercise. They performed several practice sets with light loads for all exercises (e.g. bench press, leg press, etc.) to ensure proper form and safety during the subsequent 1RM testing and the 12-week training program. All participants signed a written informed consent form and completed the Physical Activity Readiness Questionnaire (PAR-Q) a validated and reliable screening tool widely applied across diverse populations to assess readiness for exercise participation. Ethical approval for all study procedures was obtained from the Research and Ethics Committee of the Hakim Sabzevari University (Ethics code: IR.HSU.REC.1404.044). The trial was prospectively registered at the Iranian Registry of Clinical Trials (https://irct.behdasht.gov.ir/trial/87431) under registration number (IRCT20120129008863N14). All processes adhered to the principles outlined in the latest revision of the Declaration of Helsinki [15].

Physical assessments were performed by a qualified examiner during the initial visit. Baseline evaluations, including anthropometric measurements, body composition analysis, and biochemical assays, were subsequently conducted during a second visit. Assessments were performed at two time points: baseline and after completion of the 12-week training program. Post-intervention testing was scheduled 48 h after the final exercise session for all groups, while baseline testing occurred 48 h before initiating training and/or supplementation. All measurements were undertaken under standardized environmental conditions, in the morning, and within a 1-hour time frame to minimize diurnal variation. Body composition, including body fat percentage (PBF) and fat-free mass (FFM), was assessed using a multi-frequency bioelectrical impedance analyzer (InBody 720, Biospace, Seoul, Korea) according to the manufacturer’s instructions. The participants were asked to fast for at least 4 h and void their bladder before the measurement.

**Figure 1.**
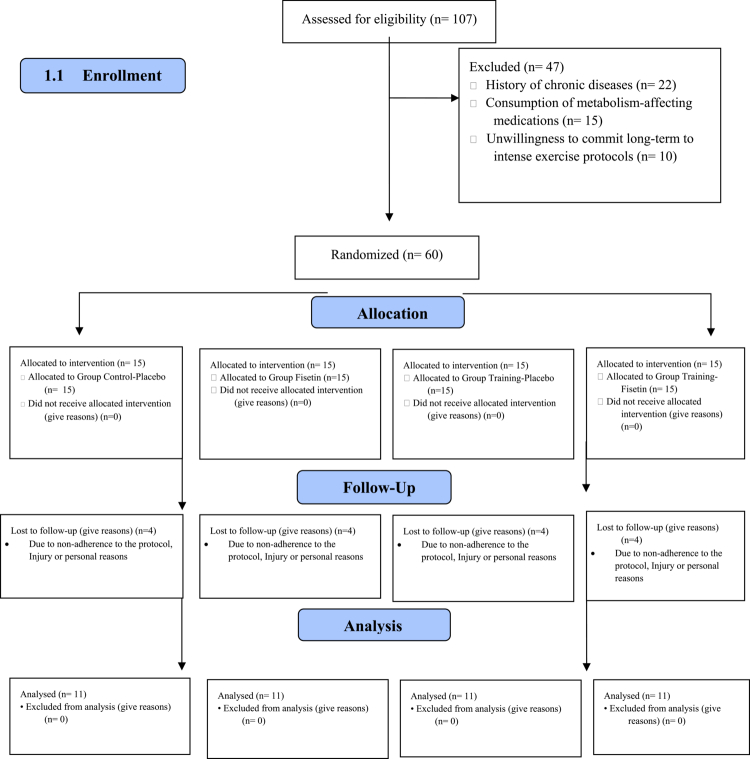
The details of the participant registration, screening, and random assignment process.

Randomization was implemented using a computer-generated block randomization list (block size = 4), ensuring equal allocation into four intervention arms: control-placebo (CP), fisetin (F) (200 mg/day), training-placebo (TP), or training-fisetin (TF) (Figure 1). Allocation concealment was achieved through the use of opaque, sealed envelopes prepared by an independent researcher who was not involved in participant recruitment or data collection. This study followed a double-blind design for the supplementation aspect, meaning participants were unaware of whether they received a placebo or fisetin. The coaches or trainers were unaware of the assignment/task of the supplementary groups.

To control for dietary influences, participants adhered to a standardized diet for 72 h prior to both baseline and final measurements. The final randomized sample included 60 participants (*n* = 15 per group). During follow-up, 16 participants withdrew from the study for the following reasons: personal scheduling conflicts (*n* = 6), minor non-training-related illnesses (*n* = 5), and a lack of perceived readiness to maintain the required exercise frequency (*n* = 5). Importantly, no adverse events or exercise-induced injuries were reported during the 12-week intervention.

Consequently, the per-protocol analysis was based on data from 44 participants (*n* = 11 per group). All attrition events and their underlying reasons were systematically documented, and participant flow was visually represented in the CONSORT diagram to ensure methodological transparency (Figure 1).

### 2.2. Training protocol

The resistance training was conducted using an interval resistance training (IRT) model. Unlike traditional circuit training, IRT involves alternating between high-intensity sets (60% 1RM for 13 repetitions) and low-intensity active recovery periods (20% 1RM for 15 repetitions) within each exercise, rather than passive rest. This model is designed to enhance metabolic demand and has been previously explored in sedentary clinical populations to improve insulin sensitivity and inflammatory profiles [16]. The detailed characteristics of the training plan are summarized in Table 1.

**Table 1.** Detailed combined training protocol (12-week intervention).

Training component	Description & parameters
I. Resistance Phase	Interval Resistance Training (IRT) Model
Exercise Sequence	1. Back Squat, 2. Bench Press, 3. Leg Curl, 4. Biceps Curl, 5. Leg Press, 6. Shoulder Press, 7. Leg Extension, 8. Lat Pulldown
Work Set (High Intensity)	3 Sets × 13 Repetitions at 60% of 1RM
Active Recovery (Low Intensity)	15 Repetitions at 20% of 1RM (immediately following each work set)
1RM Assessment	Estimated via Brzycki equation; re-assessed every 4 weeks to adjust loads.
Supervision	Directly supervised by certified exercise professionals.
II. Aerobic Phase	Treadmill Bout (immediately following resistance phase)
Weeks 1–4	15 min at 50% HRmax
Weeks 5–8	20 min at 60% HRmax
Weeks 9–12	25 min at 70% HRmax
Monitoring	Real-time monitoring via polar chest-strap heart-rate monitors.
III. Session Structure	Total duration: ~60–80 min
Warm-up	10 min (Standardized dynamic stretching/light activity)
Cool-down	10 min (Static stretching)
Frequency	3 Sessions per week

The participants’ one‑repetition maximum (1RM) was estimated using the Brzycki equation after a standardized warm‑up. Following light preparatory sets, each participant selected a load they could perform for up to 10 repetitions. If more than 10 repetitions were achieved, the load was increased after a brief rest, and the attempt was repeated until a load producing ≤10 repetitions was identified. The final load and repetition count were recorded and used to calculate 1RM as:

1RM (kg) = weight (kg)/1.0278–(0.0278 × repetitions).

The resistance program was implemented as an interval, alternating lower‑ and upper‑body exercises. Eight exercises were included in each session in the following sequence: back squat, bench press, leg curl (machine), biceps curl, leg press, barbell shoulder press, leg extension (machine), and lat pulldown. Sessions were supervised by certified exercise professionals to ensure correct technique and participant safety. To determine the appropriate workload for the resistance training sessions, a 10-repetition maximum (10 RM) test was conducted for each of the selected exercises (e.g. leg press, bench press, lat pulldown). The 1RM for each movement was then estimated using the Brzycki formula. To account for strength adaptations, these assessments were repeated every four weeks, and the training loads were adjusted accordingly to maintain the prescribed relative intensity.

Every session for the training groups included 8 exercises with 60% of 1 RM, and 13 repetitions that were followed by an active rest of 20% of 1RM and 15 repetitions; participants performed each exercise in 3 sets. Every 4 weeks, each participant’s 1RM was reassessed, and training loads for the subsequent 4‑week mesocycle were adjusted according to the updated 1RM [16].

Immediately following the interval‑resistance circuit, participants performed a treadmill aerobic bout with progressive intensity and duration across the 12‑week intervention: weeks 1–4: 15 min at 50% HRmax; weeks 5–8: 20 min at 60% HRmax; and weeks 9–12: 25 min at 70% HRmax. The maximum heart rate (HRmax) was estimated using the age‑predicted equation (HRmax = 220 − age). Aerobic exercise intensity was prescribed relative to the estimated HRmax and continuously monitored in real time using Polar chest‑strap heart‑rate monitors; heart‑rate data were recorded, and workloads were adjusted as needed to maintain the target intensities.

Each session began with a 10‑min standardized warm‑up and concluded with a 10‑min cool‑down. Adherence, session attendance, and any adverse events were documented throughout the study. All the sessions were conducted under direct supervision to maximize safety and protocol fidelity.

The control participants were instructed to maintain their usual daily activities and to refrain from initiating any new structured exercise programs during the 12‑week period. All participants provided informed consent and were free to withdraw at any time. The study procedures conformed to relevant ethical guidelines, and any deviations or adverse events were recorded and reported.

Training loads were updated every 4 weeks to maintain the relative intensity of 60% and 20% 1RM despite strength gains.

### 2.3. Supplementation protocol

Participants in the supplementation arm were administered 200  mg of fisetin (Novusetin™, USA) in capsule form, taken once daily immediately after breakfast [17]. The selection of this 200 mg dosage was based on prior clinical evidence demonstrating its efficacy in significantly reducing systemic inflammatory markers and improving metabolic homeostasis in human subjects without any reported adverse effects [18]. The placebo group received identical capsules in terms of size, shape, and color, containing 200 mg of corn starch, thereby preserving the visual and tactile similarity essential for study blinding.

To minimize potential dietary confounders, all participants completed a three-day dietary recall at two critical time points: during the three days preceding the baseline blood sampling, and again in the three days prior to post-intervention blood collection. This approach facilitated the monitoring of dietary patterns and ensured that variations in energy or nutrient intake did not influence the biochemical outcomes under investigation. To minimize potential dietary confounders, participants were instructed to avoid initiating any new dietary supplements (e.g. omega-3, vitamins C and E, or herbal extracts) and to refrain from significant changes in their consumption of anti-inflammatory or antioxidant-rich foods during the 12-week intervention. These factors were monitored using 3-day dietary recalls to ensure that nutritional intake remained stable, thereby ensuring that the observed changes in biochemical markers were primarily attributable to the prescribed intervention rather than dietary fluctuations.

### 2.4. Dietary monitoring and analysis

Throughout the 12-week experimental period, all participants documented their typical dietary intake using 3-day food logs 2 weekdays and 1 weekend day collected at both baseline and at the study endpoint. These records were analyzed with Diet Analysis Plus v.10 software (Cengage Learning, Boston, MA, USA), which facilitated estimation of daily total caloric intake alongside macronutrient distribution, including carbohydrate, fat, and protein consumption (Table 2).

**Table 2.** Mean (±SD) daily nutrient intake in study groups before and after the intervention.

	CP		F		TP		TF	
Variable	Pre	post	Pre	post	Pre	post	Pre	post
Energy (kcal/day)	2240 ± 72	2243 ± 79	2265 ± 95	2258 ± 91	2238 ± 105	2235 ± 98	2252 ± 107	2245 ± 102
CHO (g/day)	278 ± 14.1	279 ± 13.9	275 ± 15.6	274 ± 14.7	281 ± 16.5	280 ± 15.4	283 ± 17.2	281 ± 16.0
Fat (g/day)	80.5 ± 9.8	80.2 ± 9.7	82.1 ± 10.1	81.8 ± 9.4	79.9 ± 10.6	79.7 ± 10.0	80.6 ± 10.3	80.3 ± 9.8
Protein (g/day)	102 ± 11.3	102.5 ± 11.7	101 ± 12.0	100.8 ± 11.5	103.1 ± 12.6	102.8 ± 12.4	103.3 ± 13.0	102.9 ± 12.7

Control-Placebo (CP), fisetin (F), Training-Placebo (TP), or Training -Fisetin (TF).

### 2.5. Blood biomarkers

Blood specimens were obtained from the right antecubital vein after a 12-h overnight fast, both 48 h prior to the first session and 48 h following the final session. The samples were collected in EDTA-coated vacutainer tubes, centrifuged for 10 min at 3000 rpm, and stored at –70 °C. All measurements were conducted between 8:00 and 10:00 am to control for circadian variation. Plasma glucose concentrations were determined via a colorimetric enzymatic assay (Parsazmun, Tehran, Iran) with a sensitivity of 5 mg/dL, while insulin resistance was quantified using the HOMA-IR formula. Plasma insulin levels were assessed through a competitive ELISA (Demeditec, Germany) with an intra-assay CV of 5.1% to 8.4%. For inflammatory markers, plasma IL-6 was analyzed using a sandwich ELISA (Biovendor, Czech Republic) with a sensitivity of 0.65  pg/mL, and TNF-*α* concentrations were measured employing an ELISA kit (Elabscience Biotechnology, Wuhan, China) with a sensitivity of 4.69  pg/mL. Finally, MaR1 levels were determined via ELISA (Sunred Biotechnology, Shanghai, China) using a CLARIOstar PLUS microplate reader, with an analytical range of 7.5–2000 pg/mL and a sensitivity of 7.247  pg/mL.

### 2.6. Statistical analysis

The adherence rate to the exercise program was quantified by taking the ratio of the number of completed sessions to the total number of available sessions, multiplied by 100. We conducted a priori calculation of the sample size utilizing the G*Power analysis software [19]. Our rationale for sample size was according to prior data [20,21], and we estimated that 11 participants (44 total) per group would provide 80% power (two-sided *α* = 0.05) to detect 7% changes in IL-6. However, we attempted to recruit 20% extra participants (10 extra participants) to account for any potential attrition. An a priori power analysis indicated that a total of 40 participants would provide 80% power to detect significant differences with an effect size of 0.40. Although 16 participants withdrew, the final sample of 44 ensured that the study remained adequately powered for its primary objectives.

The collected data were analyzed using SPSS software (version 26). Before the primary analyzes, the assumptions of parametric statistics were confirmed, including the normality of data distribution (assessed via the Shapiro‒Wilk test) and the homogeneity of variances (assessed via Levene's test). A one-way analysis of variance (ANOVA) was initially employed to ensure that there were no significant baseline differences among the four groups (CP, F, TP, and TF) at the pre-test stage.

Subsequently, a repeated measures ANOVA was used to evaluate changes in the outcome variables across the two measurement time points (pre-test and post-test) and among the four groups. To decompose significant Group × Time interactions, a Bonferroni post-hoc test was specifically applied to perform pairwise comparisons between groups at the post-test time point, as well as to evaluate intragroup changes (pre- to post-test). Furthermore, partial eta-squared (ηp^2^) was reported to indicate the magnitude of the observed effects (effect size). The relationships between the changes in statistically significant variables were explored using Pearson's correlation coefficients. For all analyzes, the significance threshold was established at *p* < 0.05.

## 3. Results

The characteristics of the participants in the experimental and placebo groups are shown in Table 3. The participants’ age, height, weight, BMI, BFP, and FFM did not differ at baseline.

**Table 3.** Characteristics of experimental and placebo participants in men with obesity.

Groups	Age (years)	Height (Cm)	Weight (Kg)	BMI (Kg/m2)	PBF (%)	FFM (Kg)
TP	28.58 ± 3.82	180.33 ± 4.56	105.27 ± 5.94	32.34 ± 0.67	31.39 ± 1.75	28.19 ± 1.52
TF	26.64 ± 3.41	180.72 ± 5.99	105.18 ± 5.71	32.16 ± 0.65	30.72 ± 1.40	28.77 ± 1.07
F	28.79 ± 4.04	179.54 ± 4.50	104.03 ± 5.52	32.25 ± 0.44	30.53 ± 1.63	27.75 ± 1.05
CP	26.35 ± 4.21	179.29 ± 6.60	104.88 ± 8.26	32.58 ± 0.77	31.20 ± 0.97	28.40 ± 1.31
***P*-Value**	0.331	0.921	0.974	0.474	0.493	0.302

Control-Placebo (CP), Fisetin (F), Training-Placebo (TP), or Training -Fisetin (TF), Body Mass Index (BMI), body fat percentage (PBF), and fat-free mass (FFM).

### 3.1. MaR1 levels

The analysis revealed a significant group × time interaction for MaR1 [F (3, 40) = 8.61, *p* > 0.001, ηp^2^ = 0.392], alongside a significant main effect of time [F (3, 40) = 22.21, *p* > 0.001, ηp^2^ = 0.357]. However, the group effect did not reach statistical significance [F (3, 40) = 3.48, *p* = 0.024, ηp^2^ = 0.207]. Post hoc comparisons indicated that post-intervention MaR1 levels were significantly higher in the TF group compared to the CP group (*p* = 0.033, 95% CI of the mean difference: 10.10–356.21).

Regarding intragroup adaptations, both training groups showed substantial improvements; MaR1 levels significantly increased by approximately 24.93% in the TP group (*p* > 0.001) and 38.67% in the TF group (*p* > 0.001). In contrast, no significant longitudinal changes were observed in the F group (*p* = 0.530) or CP group (*p* = 0.666) (Table 4).

**Table 4.** Changes in some inflationary factors and insulin resistance index in the research groups.

Stages								
Variables and Groups	Pre-Test M ± SD^a^	12^th^ Week M ± SD^a^	Percentage of changes	*P*-Value^b^	Time *P*-Value	Group *P*-Value	Time × Group *P*-Value	Partial eta squared
**MaR1 (pg/ml)**								
TP	709.02 ± 219.75	944.54 ± 168.04	24.93	0.001	0.003	0.024	0.034	0.392
TF	756.67 ± 160.18	1233.87 ± 163.28	38.67	0.001				
F	837.62 ± 198.67	887.12 ± 184.23	5.57	0.530				
CP	825.06 ± 179.87	789.15 ± 251.81	-4.55	0.666				
**IL-6 (pg/ml)**								
TP	1.95 ± 0.43	1.42 ± 0.25	−37.32	0.006	0.001	0.003	0.001	0.417
TF	2.17 ± 0.43	1.29 ± 0.19	−68.21	0.001				
F	2.06 ± 0.49	1.59 ± 0.16	−29.55	0.013				
CP	1.97 ± 0.33	2.16 ± 0.23	8.79	0.120				
**TNF-** ***a*** **(pg/ml)**								
TP	4.36 ± 0.63	2.85 ± 0.57	−52.98	0.001	0.001	0.001	0.001	0.411
TF	4.26 ± 0.29	2.98 ± 0.26	−42.95	0.001				
F	4.37 ± 0.55	3.21 ± 0.48	−36.13	0.002				
CP	4.32 ± 0.74	4.56 ± 0.51	5.26	0.436				
**FBS (mg/dl)**								
TP	110.00 ± 5.16	85.08 ± 5.43	−29.29	0.001	0.001	0.001	0.001	0.625
TF	112.53 ± 6.26	82.51 ± 7.71	−36.38	0.001				
F	109.71 ± 9.90	95.77 ± 4.50	−14.55	0.003				
CP	106.62 ± 9.30	105.73 ± 6.43	−0.84	0.710				
**Insulin (mU/L)**								
TP	20.13 ± 0.40	17.42 ± 0.43	−15.55	0.001	0.001	0.001	0.001	0.782
TF	20.43 ± 0.49	16.81 ± 0.55	−21.53	0.001				
F	20.10 ± 0.75	18.90 ± 0.51	−6.34	0.002				
CP	20.11 ± 0.67	20.42 ± 0.57	1.51	0.330				
**HOMA-IR**								
TP	5.46 ± 0.24	3.66 ± 0.29	−49.18	0.001	0.001	0.001	0.001	0.777
TF	5.67 ± 0.37	3.42 ± 0.34	−65.78	0.001				
F	5.44 ± 0.45	4.47 ± 0.27	−21.70	0.001				
CP	5.30 ± 0.57	5.33 ± 0.33	0.56	0.873				

Control-Placebo (CP), Fisetin (F), Training-Placebo (TP), or Training -Fisetin (TF).

^a^ Values are expressed as mean ± standard deviation.

^b^ Within-group *P*-value.

^c^ Significant at *P* < 0.05.

Correlation analysis was performed to explore the relationship between MaR1 and other physiological markers (Table 5). MaR1 exhibited weak to moderate negative correlations with inflammatory cytokines, including IL-6 (r = −0.492, *p* > 0.001) and TNF-*a* (r = −0.323, *p* = 0.032). Notably, MaR1 showed stronger and highly significant negative associations with key metabolic health indicators, specifically insulin (r = −0.579, *p* > 0.001), glucose (r = −0.548, *p* > 0.001), and HOMA-IR (r = −0.599, *p* > 0.001).

**Table 5.** Pearson correlation matrix between inflammatory and metabolic indices.

Variables	MaR1	IL-6	TNF-a	Insulin	FBS	HOMA-IR
MaR1	1					
IL-6	−0.492**	1				
TNF-a	−0.323*	0.661**	1			
Insulin	−0.597**	0.799**	0.659**	1		
FBS	−0.548**	0.646**	0.662**	0.819**	1	
HOMA-IR	−0.599**	0.752**	0.703**	0.935**	0.968**	1

^*^ : Correlation is statistically significant at the *P* < 0.05 level (two-tailed).

^**^ : Correlation is statistically significant at the *P* < 0.01 level (two-tailed).

Note: Sample size, *N* = 44.

### 3.2. IL-6

A significant group × time interaction [F (3, 40) = 9.545, *p* > 0.001, ηp^2^ = 0.417], time effect [F (3, 40) = 34.513, *p* > 0.001, ηp^2^ = 0.463], and group effect [F (3, 40) = 5.431, *p* = 0.003, ηp^2^ = 0.289] were observed for IL-6. Post hoc analysis confirmed that IL-6 levels in the TP, F, and TF groups were significantly lower than those in the CP group (*p* < 0.05).

Within-group analysis showed marked reductions: the TF group experienced the greatest decrease (68.21%, *p* > 0.001), followed by the TP group (37.32%, *p* = 0.006) and the F group (8.79%, *p* = 0.013). No significant change occurred in the CP group (*p* = 0.120).

### 3.3. TNF-*a*

TNF-*a* levels showed a significant group × time interaction [F (3, 40) = 9.30, *p* > 0.001, ηp^2^ = 0.411], as well as significant time [F (3, 40) = 50.50, *p* > 0.001, ηp^2^ = 0.558] and group effects [F (3, 40) = 17.68, *p* > 0.001, ηp^2^ = 0.570]. Post-hoc tests indicated that all intervention groups (TP, TF, and F) had significantly lower TNF-*a* levels compared to the CP group (*p* > 0.001).

Significant intragroup reductions were observed across all intervention groups (F, TP, and TF): 52.98% in TP (*p* > 0.001), 42.95% in TF (*p* > 0.001), and 36.13% in F (*p* = 0.002). The CP group showed no significant change (*p* = 0.436) (Table 4).

### 3.4. Fasting blood glucose (FBS)

Statistical analysis revealed a significant group × time interaction for FBS [F (3, 40) = 22.20, *p* > 0.001, ηp^2^ = 0.625]. Furthermore, significant main effects were observed for both time [F (3, 40) = 162.07, *p* > 0.001, ηp^2^ = 0.802] and group [F (3, 40) = 6.691, *p* > 0.001, ηp^2^ = 0.334], with a small between-group effect size (ES = 0.334). Post-hoc comparisons indicated that post-intervention FBS levels in the TF and TP groups were significantly lower than those in the CP group (*p* = 0.004).

Intragroup analysis demonstrated significant reductions in FBS across all intervention groups: 29.29% in the TP group (*p* > 0.001), 36.38% in the TF group (*p* > 0.001), and 14.55% in the F group (*p* = 0.003). No significant changes were observed in the CP group (*p* = 0.710) (Table 4).

### 3.5. Fasting insulin

A significant group × time interaction was found for fasting insulin [F (3, 40) = 47.78, *p* > 0.001, ηp^2^ = 0.782]. Significant effects were also noted for time [F (3, 40) = 208.777, *p* > 0.001, ηp^2^ = 0.839] and group [F (3, 40) = 43.619, *p* > 0.001, ηp^2^ = 0.766], with a large between-group effect size (ES = 0.766). Post-hoc tests confirmed that insulin levels in the TP, TF, and F groups were significantly reduced compared to the CP group (*p* > 0.001).

Within-group changes showed significant improvements in insulin sensitivity, with reductions of 15.55% in the TP group (*p* > 0.001), 21.53% in the TF group (*p* > 0.001), and 6.34% in the F group (*p* = 0.002). The CP group showed no significant longitudinal change (*p* = 0.330) (Table 4).

### 3.6. HOMA-IR

The analysis for HOMA-IR indicated a significant group × time interaction [F (3, 40) = 46.397, *p* > 0.001, ηp^2^ = 0.777]. Significant main effects were observed for time [F (3, 40) = 287.606, *p* > 0.001, ηp^2^ = 0.878] and group [F (3, 40) = 17.791, *p* > 0.001, ηp^2^ = 0.572], with a large effect size (ES = 0.572). Post hoc analysis showed that HOMA-IR levels in all intervention groups (TP, TF, and F) were significantly lower than those in the CP group (*p* < 0.05).

The most pronounced intragroup improvements were seen in the training groups (TP and TF); HOMA-IR decreased by 49.18% in the TP group (*p* > 0.001) and 65.78% in the TF group (*p* > 0.001). The F group also showed a significant reduction of 21.70% (*p* > 0.001), whereas no significant variation was found in the CP group (*p* = 0.873) (Table 4).

Moving beyond single correlations, our analysis confirmed the expected strong internal relationships within both the inflammatory and metabolic groups. A very strong positive correlation (*r* = 0.661, *p* < 0.001) was observed between the two primary inflammatory markers, IL-6 and TNF-*a*. Similarly, the variables associated with insulin resistance and glucose regulation, insulin, glucose, and HOMA-IR, were highly intercorrelated. Specifically, HOMA-IR demonstrated an exceptionally strong positive relationship with both insulin (*r* = 0.935, *p* < 0.001) and glucose (*r* = 0.968, *p* < 0.001), further underscoring the validity of these markers. The strong positive correlation between insulin and glucose (*r* = 0.819, *p* < 0.001) also affirms the tight regulatory loop of the intervention blood sugar. Finally, and most notably, the analysis revealed strong inter-group relationships connecting inflammation and insulin resistance. Both inflammatory markers, IL-6 and TNF-*a* exhibited significant positive correlations with all metabolic markers. IL-6 showed strong associations with insulin (*r* = 0.799), glucose (*r* = 0.662), and HOMA-IR (*r* = 0.752), while TNF-*a* was similarly correlated with insulin (*r* = 0.659), glucose (*r* = 0.703), and HOMA-IR (r = 0.703) (Table 5). Collectively, these results unequivocally demonstrate that increased levels of inflammatory mediators are strongly and positively correlated with a significant rise in insulin, glucose, and overall HOMA-IR.

## 4. Discussion

Our results clearly show that pairing combined aerobic–resistance training with fisetin supplementation leads to measurable and clinically relevant improvements in inflammation resolution and metabolic control among obese men. Plasma MaR1 concentrations increased significantly in the TP and TF groups, while IL-6, TNF-*α*, fasting blood glucose, insulin, and HOMA-IR levels decreased markedly in all active intervention arms compared to the CP group. These outcomes can be explained by an interaction between training-induced physiological adaptations and the molecular actions of fisetin. To our knowledge, no randomized human trial has previously examined whether fisetin can modulate MaR1 or other specialized pro-resolving mediators; therefore, our study directly addresses this unresolved question by pairing fisetin with a structured exercise program. The increase in MaR1 observed in our TP and TF groups is consistent with its known synthesis pathway from docosahexaenoic acid (DHA) via 12‑lipoxygenase [22], a process that can be stimulated by regular exercise through enhanced DHA mobilization and the upregulation of lipid mediator enzymes [23–26].

Repeated mechanical stimuli from hybrid resistance–aerobic training promote macrophage polarization toward the anti-inflammatory M2 phenotype. This shift creates a cellular environment conducive to the biosynthesis of specialized pro-resolving mediators such as MaR1 [27,28]. The reduction in systemic inflammatory markers (IL-6, TNF-*α*) further lowers the inhibitory burden on resolution-phase pathways, allowing for efficient synthesis and release of MaR1 [29,30]. Because this is the first exercise intervention to document an upregulation of MaR1 in humans, no established mechanistic framework currently exists. We speculate that repetitive mechanical loading, improved DHA mobilization [23–26], and enhanced activity of 12/15‑lipoxygenase could converge to favor MaR1 biosynthesis [31]. Still, targeted molecular assays (e.g. DHA flux, lipoxygenase expression, and macrophage polarization) are required to verify these pathways.

Exercise suppresses NF-κB signaling and downregulates toll-like receptor activation, reducing the transcription and secretion of pro-inflammatory cytokines from muscle and immune cells [32]. It also enhances mitochondrial function, reduces ectopic lipid deposition in muscle and liver, and improves insulin receptor signaling [33,34]. These changes collectively diminish lipotoxicity-driven inflammation and improve glucose transport via increased GLUT-4 translocation.

The marked reductions in IL‑6 and TNF‑α likely reflect a dual mechanism: the immunomodulatory effects of structured training and the bioactivity of fisetin. Together, these factors appear to suppress NF‑κB signaling, reduce oxidative stress, and preserve insulin receptor function changes that align closely with the improvements we observed in HOMA‑IR. [35–37]. Additionally, fisetin promotes M2 macrophage polarization and preserves lipoxygenase activity, protecting the structural integrity of membrane phospholipids from oxidative damage [17,38], thus ensuring substrate availability for MaR1 synthesis. Through the suppression of cytokine burden and safeguarding of metabolic signaling pathways, fisetin amplifies the beneficial metabolic and immune shifts initiated by exercise. The absence of a significant change in MaR1 in the fisetin-only group may indicate that supplementation alone is insufficient to stimulate SPM pathways without the mechanical and metabolic stimuli provided by exercise. It is important to acknowledge the dual nature of exercise-induced inflammation. While chronic exercise is a potent anti-inflammatory intervention, acute bouts of high-intensity training can initially provoke a transient pro-inflammatory response, characterized by elevations in cytokines such as IL-6. This is particularly relevant in exercise-naive populations, such as the obese men in the current study, whose systems may be more susceptible to the mechanical and metabolic stress of new training stimuli. However, our findings indicate that 12 weeks of regular combined training, especially when augmented by fisetin, promotes a systemic shift toward the resolution of inflammation (pro-resolving phase), likely through the chronic upregulation of specialized pro-resolving mediators such as MaR1.

In our cohort, the combination of exercise and fisetin not only increased MaR1 and shifted macrophages toward an anti‑inflammatory profile but also translated into tangible metabolic benefits, including improved insulin sensitivity and better glucose control. The 38.67% increase in MaR1 in the TF group aligns with the hypothesis that exercise-derived DHA mobilization is enhanced when oxidative stress is reduced by fisetin. At the same time, it dampens chronic inflammatory signaling through the suppression of IL‑6 and TNF‑α production, thereby reducing the systemic inflammatory burden. In addition, this synergy enhances insulin sensitivity and supports better glycemic control by preserving insulin receptor functionality, facilitating greater glucose uptake into skeletal muscle, and mitigating metabolic stress. Together, these processes reflect a coordinated improvement in immune regulation and metabolic health, underscoring the therapeutic potential of combining structured exercise with targeted flavonoid supplementation in obesity management.

**Figure 2.**
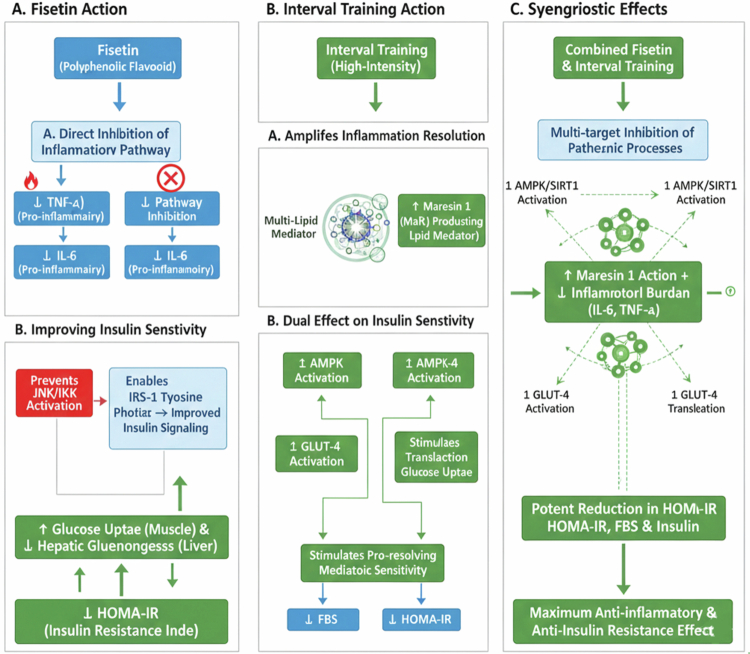
Synergistic action of fisetin and interval training on anti-inflammatory and metabolic pathways.

Taken together, these adaptations may translate into tangible benefits for obese patients aiming to reduce inflammation and regain metabolic control (Figure 2).

Among the limitations of the present study is the fact that it was conducted on a relatively small and homogeneous sample of 44 obese adult men from a single geographic region, which may restrict the generalizability of the results to other age groups, women, or populations with different ethnic and genetic backgrounds. Consequently, future investigations with larger and more diverse cohorts are needed to confirm the wider applicability of these findings. Moreover, physiological and biochemical parameters were evaluated only immediately after completion of the 12-week intervention, leaving it unclear whether the observed benefits, such as increased MaR1 concentration, reduced pro-inflammatory cytokines, and improved insulin sensitivity, are sustained over time.

Several limitations of the present study warrant consideration. First, the generalizability of our findings is constrained by the recruitment of a relatively homogeneous cohort of inactive obese men from a single geographic region; therefore, these results may not be directly applicable to women, older populations, or individuals from diverse ethnic backgrounds. Future multicenter studies involving more diverse populations are necessary to confirm these preliminary findings. Second, our investigation relied on systemic biochemical markers rather than direct intracellular molecular assessments, such as NF-κB signaling or macrophage polarization markers within adipose tissue. Furthermore, the absence of a long-term follow-up period post-intervention limits our understanding of the sustainability of the observed improvements. Future research should incorporate tissue biopsies and longitudinal assessments to further elucidate the underlying mechanisms and their long-term clinical relevance. Finally, while BMI was utilized for initial group allocation, we acknowledge its inherent limitations in distinguishing between fat and muscle mass. However, the sedentary nature of our cohort and the consistent baseline body fat percentage data mitigate the potential risk of participant misclassification.

In summary, this study shows that twelve weeks of combined interval resistance and aerobic training, particularly when paired with fisetin supplementation, can lead to meaningful improvements in metabolic and inflammatory profiles in obese men. The intervention increased the levels of MaR1, a key pro-resolving mediator, while reducing IL-6, TNF-*α*, fasting glucose, insulin, and HOMA-IR, indicating enhanced inflammation resolution and better insulin sensitivity. The synergy between training-induced physiological adaptations and fisetin’s anti-inflammatory and antioxidant properties appears to restore immune balance while supporting glycemic control. These findings suggest that integrating structured physical training with targeted nutritional supplementation could be a promising approach for reducing obesity-related metabolic risk. While the results are encouraging, further studies with larger and more diverse samples, longer follow-up, and direct molecular evaluations are warranted to fully confirm the clinical relevance and underlying mechanisms of these adaptations.

## Data Availability

The data that support the findings of this study are available from the corresponding author, [a.saedi@uok.ac.ir], upon reasonable request.
